# Retraction Note: The lincRNA-ROR/miR-145 axis promotes invasion and metastasis in hepatocellular carcinoma via induction of epithelial-mesenchymal transition by targeting ZEB2

**DOI:** 10.1038/s41598-023-32251-x

**Published:** 2023-03-29

**Authors:** Chen Li, Lu Lu, Bing Feng, Kai Zhang, Siqi Han, Daorong Hou, Longbang Chen, Xiaoyuan Chu, Rui Wang

**Affiliations:** 1 Department of Medical Oncology, Jinling Hospital, Medical School of Nanjing University, Nanjing, 210002 Jiangsu Province China; 2 Animal Core Facility of Nanjing Medical University, Nanjing, Jiangsu China

Retraction of: *Scientific Reports* https://doi.org/10.1038/s41598-017-04113-w, published online 05 July 2017

The Editors have retracted this Article.

After publication of this Article the Editors were alerted to concerns with a number of figures, specifically:

- Figure 2D, HepG2 the “Vector” condition “Migration” and “Invasion” appear to partially overlap;

- Figure 2D, HCCLM3 “siCtrl, Migration” condition appears to partially overlap with Figure 6C HCCLM3 “miR-NC/mimics Migration”;

- Figure 2D, HCCLM3 “siCtrl, Invasion” condition seem to partially overlap with Figure 6C HCCLM3 “miR-NC/mimics Invasion”, as well as with Figure 5B HCCLM3 “miR-145/inhibitor, Invasion”;

- Figure 6C, HepG2 the “miR-NC/inhibitor” condition “Migration” and “Invasion” appear to partially overlap;

- GAPDH western blot in Fig 3C appears to be identical as GAPDH in Fig 5D HCCLM3 cell line;

- Figure 5D, the GAPDH western blot panel of the HCCLM3 cell line appears to be different from the provided data;

- Figure 5E, the HepG2 E-cadherin miR-NC/inhibitor and the vimentin miR-145/inhibitor panels appear to be similar;

- Figure 5E, the HepG2 N-cadherin and vimentin miR-145/inhibitor panels appear to be different from the provided data;

- Figure 5E, The 1st and 3rd panel of HepG2 N-cadherin miR-145/inhibitor appear to be similar to the HepG2 vimentin miR-NC/mimics panels in Fig 6D;

- Figure 6D, HepG2 vimentin miR-NC/mimics 1st panel appears to be different from the provided data;

- Figure 6D, HCCLM3 beta-catenin and N-cadherin miR-145/inhibitor appears to be different from the provided data.

The Authors provided data to address these issues but are unable to provide the original raw data underlying these figures due to the time that has passed since publication. The Editors therefore no longer have confidence in the results reported in this Article.

All Authors agree with the retraction.

